# Melanic Cancer of the Cheek

**Published:** 1826-07

**Authors:** 


23. *Melanic Cancer of the Cheek.* M. Lisfranc lately removed, with success for the present, a tumour of this kind, developed in the cheek of an aged person, a native of Belgrade. The tumour had been only a year in forming, and was seated on the buccinator and masseter muscles, extending from the zigomatic arch to the inferior edge of the lower jaw. The skin covering this tumour was of a violet hue, and the submaxillary glands were swelled. It was considerably moveable, but still adherent to the subjacent muscles and other tissues. It occasioned violent and lancinating pains. It was removed by two elliptic incisions, and was found to consist of a substance in cells, as black as ink.

Some days after the operation, both swelling and lancinating pains returned in the part, and here the chief merit of the case lies. Leeches were daily employed, with the greatest perseverance, and at length the whole of the bad symptoms disappeared, the wound healed, and the patient was discharged from La Pitié cured.

We believe that many relapses of cancer take place from want of attention to the subsequent treatment when an operation is performed. The first dawnings of returning pain should be met by leeches in great number, so as to deprive the parts of that blood from which the morbid process derives the pabulum with which it constructs its baleful work.

